# Sorafenib inhibits ovarian cancer cell proliferation and mobility and induces radiosensitivity by targeting the tumor cell epithelial–mesenchymal transition

**DOI:** 10.1515/biol-2022-0066

**Published:** 2022-06-15

**Authors:** Chuntao Tian, Ying Liu, Lingfei Xue, Dong Zhang, Xiaotong Zhang, Jing Su, Jiaohong Chen, Xiangke Li, Liuxing Wang, Shunchang Jiao

**Affiliations:** Department of Oncology, The Sanmenxia Central Hospital, Henan 472000, China; Department of Pathology, The Sanmenxia Central Hospital, Henan 472000, China; Department of Oncology, The First Affiliated Hospital, Zhengzhou University, Zhengzhou 450052, China; Department of Oncology, The General Hospital of Chinese PLA, Beijing 100853, China

**Keywords:** ovarian cancer, sorafenib, transforming growth factor-β1, radiosensitivity

## Abstract

Sorafenib, a pan-protein kinase inhibitor, inhibits the activity of various kinases (like vascular endothelial growth factor, platelet-derived growth factor, and rapidly accelerated fibrosarcoma) and clinically has been used to treat different human cancers. This study investigated its antitumor activity in ovarian cancer and the underlying molecular events. To achieve that, ovarian cancer SKOV-3 cells were treated with or without sorafenib (10 µM), transforming growth factor (TGF)-β1 (10 ng/mL), sorafenib (10 µM) + TGF-β1 (10 ng/mL), and TGF-β1 (10 ng/mL) + Ly2157299 (5 µM), followed by 8-Gy radiation. The cells were then subjected to cell viability, wound healing, Transwell, caspase-3 activity, and western blot assays. TGF-β1 treatment enhanced ovarian cancer cell epithelial–mesenchymal transition (EMT), whereas sorafenib and a selective TGF-β1 inhibitor Ly2157299 reversed tumor cell EMT, invasion, and expression of EMT markers (E-cadherin and vimentin). Sorafenib and Ly2157299 treatment also significantly reduced the tumor cell viability. Furthermore, both sorafenib and Ly2157299 significantly enhanced ovarian cancer cell radiosensitivity, as assessed by a caspase-3 activity assay. In conclusion, sorafenib inhibited ovarian cancer cell proliferation and mobility and induced tumor cell radiosensitivity. Molecularly, sorafenib could inhibit the TGF-β1-mediated EMT. Future studies will assess sorafenib anti-ovarian cancer activity plus TGF-β1 inhibitors in ovarian cancer *in vivo*.

## 1 Introduction

Ovarian cancer is still a worldwide health burden in women, accounting for more than 295,000 new cases and 184,000 cancer-related deaths globally in 2018 [1]. In China, ovarian cancer ranked tenth in terms of cancer incidence and ninth in terms of mortality in 2015, although ovarian cancer cases have been decreasing over the last decade [2]. Clinically, ovarian cancer is usually diagnosed at the late stage as an aggressive disease [3]. Treatment for ovarian cancer depends on the stage, but surgery combined with platinum-based chemoradiotherapy is regarded as the standard therapy for ovarian cancer patients, regardless of the histological subtype. Meanwhile, radiation, hormone, and immune therapies are also used to control ovarian cancer [4,5,6,7,8,9]. However, up to 80% of the patients will eventually develop drug resistance, leading to disease relapse and progression. Effective treatments for platinum-resistant ovarian cancer are still lacking due to the low response rate to non-platinum-based drugs (such as topotecan, etoposide, and pazopanib). Moreover, targeted therapy with bevacizumab (a representative antiangiogenesis drug), poly-ADP ribose polymerase (PARP) inhibitors, and multidisciplinary treatment programs have shown a better control of advanced ovarian cancer [7]. For example, olaparib, a PARP inhibitor, has been demonstrated to have beneficial effects against adult BRCA-mutated advanced ovarian cancer [10] and was approved by the US Federal Drug Administration to treat ovarian cancer in 2014 [11]. Thus, the continuous search for and identification of novel agents to control ovarian cancer as well as a better understanding of the pathogenesis and molecular mechanisms of this deadly disease are urgently needed now.

Transforming growth factors (TGFs) are polypeptide-related cell growth mediators. To date, there are two types of TGFs: TGF-α and TGF-β; each can be further classified into different subtypes, such as TGF-β1, which has different tissue distributions and functions in cells [12]. TGF-β1 is important for embryonic development, homeostasis of cell physiology, and tissue response to injury [13]. During tumorigenesis and cancer progression, TGF-β1 is considered to be an important cytokine for the induction of tumor cell epithelial-to-mesenchymal transition (EMT) [14]. Thus, it is frequently used as a stimulant in the EMT cell model in medical research [15,16]. EMT shows that the epithelia lose their cell polarity and adhesion capacity but gain their cell mobility ability, i.e., the mesenchymal stem cell property. EMT is also the hallmark of tumor development and progression. TGF-β1 is involved in ovarian tumorigenesis, while ovarian cancer cell EMT and metastasis have been associated with chemoresistance and poor survival of ovarian cancer patients [17,18,19,20,21]. Moreover, TGF-β can inhibit the growth of the ovarian surface epithelial cells and the early stage of ovarian cancer cells, but it also promotes the tumor growth of ovarian cancer at the later stages [17]. In addition, the expression of TGF-β1 and its receptors is associated with ovarian cancer biological features and sensitivity to paclitaxel/carboplatin treatment [22]. Thus, targeting TGF-β1 activity could be an effective means to control ovarian cancer.

Sorafenib toluene sulfonate (sorafenib) is a pan-protein kinase inhibitor that suppresses the activity of various kinases, such as vascular endothelial growth factor (VEGF), platelet-derived growth factor (PDGF), and RAF. This broad-spectrum antitumor drug was recently co-developed by Bayer and ONYX Pharmaceuticals Co. It not only inhibits the RAF/MEK/ERK signaling pathway but also activates VEGF1/2/3, PDGF, FMS-like tyrosine kinase 3 (FLT-3), and c-Kit [23,24,25]. Sorafenib is able to block TGF-β1-mediated tumor cell EMT; thus, it possesses a dual antitumor effect by inhibiting both tumor cell proliferation and angiogenesis [26]. Since 2007 [27], sorafenib has been approved in more than 100 countries to treat thyroid, renal, and hepatocellular carcinoma. In this study, we assessed the activity of sorafenib in the regulation of TGF-β1-mediated ovarian cancer cell proliferation, mobility, and radiosensitivity *in vitro*. We expect that the *in vitro* data will support the clinical application of sorafenib for the treatment of ovarian cancer.

## 2 Materials and methods

### 2.1 Cell line and culture

Human ovarian cancer SKOV-3 cells were obtained from the Shanghai Institute of Cell Biology (China) and maintained in Dulbecco’s modified Eagle’s medium (DMEM; Gibco, Gaithersburg, MD, USA) supplemented with 10% fetal bovine serum (FBS; Gibco) in a CO_2_ incubator at 37°C. The cells were passed once every 2–4 days.

There were 5 cell treatment groups (*n* = 3 per cell treatment group), i.e., SKOV-3 cell control, TGF-β1 (10 ng/mL)-alone treatment according to previous studies [28,29,30], sorafenib (10 µM)-alone, sorafenib (10 µM) + TGF-β1 (10 ng/mL), and TGF-β1 (10 ng/mL) + Ly2157299 (a selective TGF-β1 inhibitor, 5 µM) group. Human TGF-β1 was obtained from Pepro Tech (Shuzhou, China), while sorafenib and Ly2157299 were from TargetMol (Shanghai, China).

### 2.2 Western blot

Western blot was performed according to a previous study [31]. The total cellular protein was extracted using a protein extraction kit (AmyJet Scientific, Wuhan, China), and the protein concentration was measured using a bicinchoninic acid protein assay kit (CWBIO, Beijing, China), according to the manufacturer’s protocols. Next the protein samples underwent sodium dodecyl sulfate-polyacrylamide gel electrophoresis and were transferred onto polyvinylidene difluoride membranes (Millipore, Billerica, MA, USA). The membranes were first incubated in 5% skim milk solution at 4°C overnight for western blotting. Next these membranes were incubated with a primary antibody against vimentin or E-cadherin from Cell Signaling Technology (Danvers, MA, USA), both at a dilution of 1:1,000, at room temperature for 2 h. After that, the membranes were washed in phosphate-buffered saline (PBS)-Tween 20 (PBS-T) three times and then incubated with a secondary antibody (AmyJet Scientific, Wuhan, China) at room temperature for 1 h. Finally, the membranes were treated with enhanced chemiluminescence reagents (Amersham Biosciences, Shanghai, China), detected by using the Bio-Rad gel imaging system, and quantified using Quantity One software (Bio-Rad, Hercules, CA, USA).

### 2.3 Cell viability assay

The cell viability assay was conducted using the 3-(4,5-dimethylthiazol-2)-2,5-diphenyltetrazolium bromide (MTT) assay, according to a previous study [32]. In brief, ovarian cancer SKOV-3 cells in the logarithmic growth phase were passaged using trypsin digestion and reseeded into a 96-well plate at a density of 5,000 cells per well and cultured overnight. Next 6 wells of cells for each treatment group (*n* = 3 per cell treatment group) were treated with control; TGF-β1 (10 ng/mL) alone, according to previous studies [28,29,30]; sorafenib (10 µM) alone; sorafenib (10 µM) + TGF-β1 (10 ng/mL); and TGF-β1 (10 ng/mL) + Ly2157299 (a selective TGF-β1 inhibitor, 5 µM) for up to 72 h. After that, 10 µL of MTT (Shanghai Beyotime Biological Technology Co., Ltd, Shanghai, China) was added to each well of cell culture, and the cells were further incubated for 4 h. The optical density was measured using a spectrophotometer at 560/590 nm, according to the manufacturer’s instructions.

### 2.4 Tumor cell wound healing and Transwell assays

For the wound healing assay, ovarian cancer SKOV-3 cells in the logarithmic growth phase were reseeded into 6-well plates at a density of 3 × 10^5^ cells per well and grown for 2 days to reach approximately 95% cell confluency. After that, the cells were wounded using a 200-µL pipette tip, washed with ice-cold PBS 3 times, and cultured for up to 72 h. Then, photographs of the cells were taken under an inverted microscope, and the images were quantified.

For the Transwell assay, SKOV-3 cells were seeded into Transwell chambers (Corning, Corning, NY, USA) at a density of 1 × 10^5^ cells per chamber (the Transwell chamber filter was precoated with 50 µL of Matrigel by the manufacturer), while the bottom chambers were filled with 200 µL of DMEM containing 20% FBS. The Transwell chambers were then cultured at 37°C for 24 h. At the end of the experiment, the cells on top of the filter were wiped off using a cotton swab, while the cells that invaded the reverse side of the filter were fixed for 10 min in 10% formalin and then stained for 10 min in 0.5% crystal violet. The number of cells on the filter was counted under an inverted microscope for five randomly selected microscopic fields at a magnification of 400×. The assay was performed in duplicate and repeated three times.

### 2.5 Tumor cell radiosensitivity assay

SKOV-3 cells after each treatment (*n* = 3 per cell treatment group) were placed under a linear accelerator 6MV-X-ray (2100EX, Varian, Palo Alto, CA, USA) at room temperature, covered with tissue glue, and subjected to full-field irradiation as uniform as possible with an irradiation dose of 8 Gy once. The absorbed dose rate was fixed at 300 cGy/min, and the irradiation distance was set at 100 cm, according to previous studies [33,34,35]. After that, the cells were collected and made into suspensions, and then 400 cells were added to 24-well culture dishes containing 1% agarose (Gibco). The cells were then grown in a humidified incubator with 5% CO_2_ at 37°C for 14 days, with the medium replaced with a fresh medium every 3 days. Cell colonies containing 50 cells or more were counted under an inverted light microscope, and the caspase-3 activity was assayed using a caspase-3 activity kit from Beyotime (Beijing, China), according to the kit’s instructions.

### 2.6 Statistical analysis

Our experiments were repeated at least three times and statistically analyzed using GraphPad Prism 6.0 software (GraphPad Software, La Jolla, CA, USA) with the chi-squared test to determine the difference between two independent variables, bi-categorical variables, or nonparametric analysis to assess the difference between two groups. A value of *P* < 0.05 was considered statistically significant.

## 3 Results

### 3.1 TGF-β1 enhanced ovarian cancer cell EMT, but sorafenib and Ly2157299 treatment reversed it

**Figure 1:**
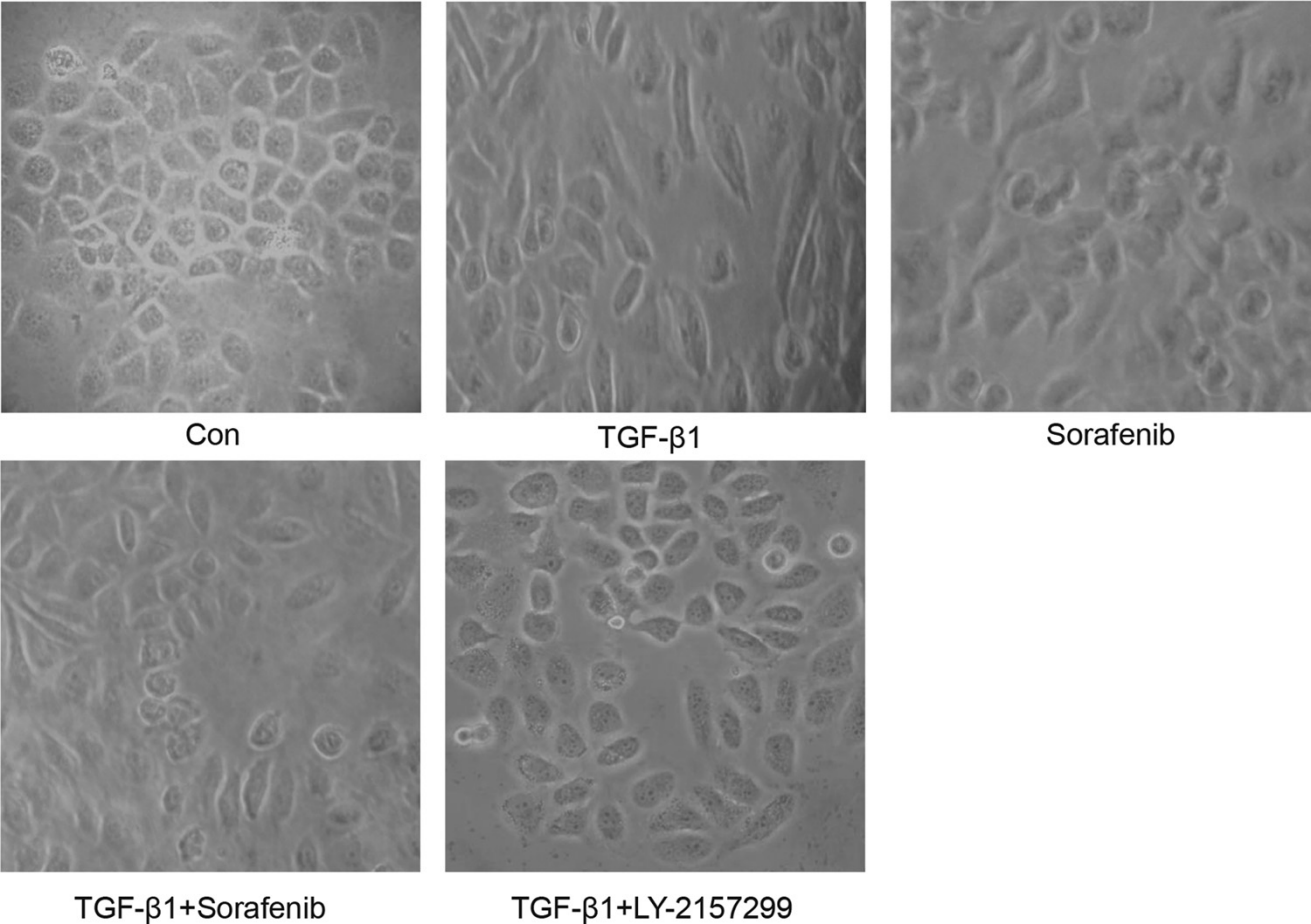
Effects of sorafenib on the regulation of TGF-β1-induced changes in ovarian cancer cell morphology. SKOV-3 cells were cultured and treated in triplicate with 10 µM sorafenib, 10 ng/mL TGF-β1, 10 µM sorafenib + 10 ng/mL TGF-β1, and 10 ng/mL TGF-β1 + 5 µM Ly2157299 for 48 h, and the cells were photographed under an inverted light microscope to assess the changes in cell morphology.

In the current study, we first repeated the TGF-β1-mediated ovarian cancer cell EMT model *in vitro*. After 48 h of cell culture, we observed the morphology under an inverted microscope. Specifically, in the control group, the SKOV-3 cells showed a normal growth pattern and tight intercellular connections with irregular polygonal morphology. In the TGF-β1 group, a portion of the SKOV-3 cells lost their normal structure by forming obvious spindles, fibroblast-like cells, while the connection between the cells was observed to be more loose and messy. Moreover, the cells after sorafenib-alone treatment showed more irregularities compared to those of the control group, but these cells did not form spindle-like cells and the intercellular connections started to be lost. However, the cells after TGF-β1 + sorafenib and TGF-β1 + Ly2157299 treatment showed a morphology that was similar to that of the control group (Figure 1).

**Figure 2:**
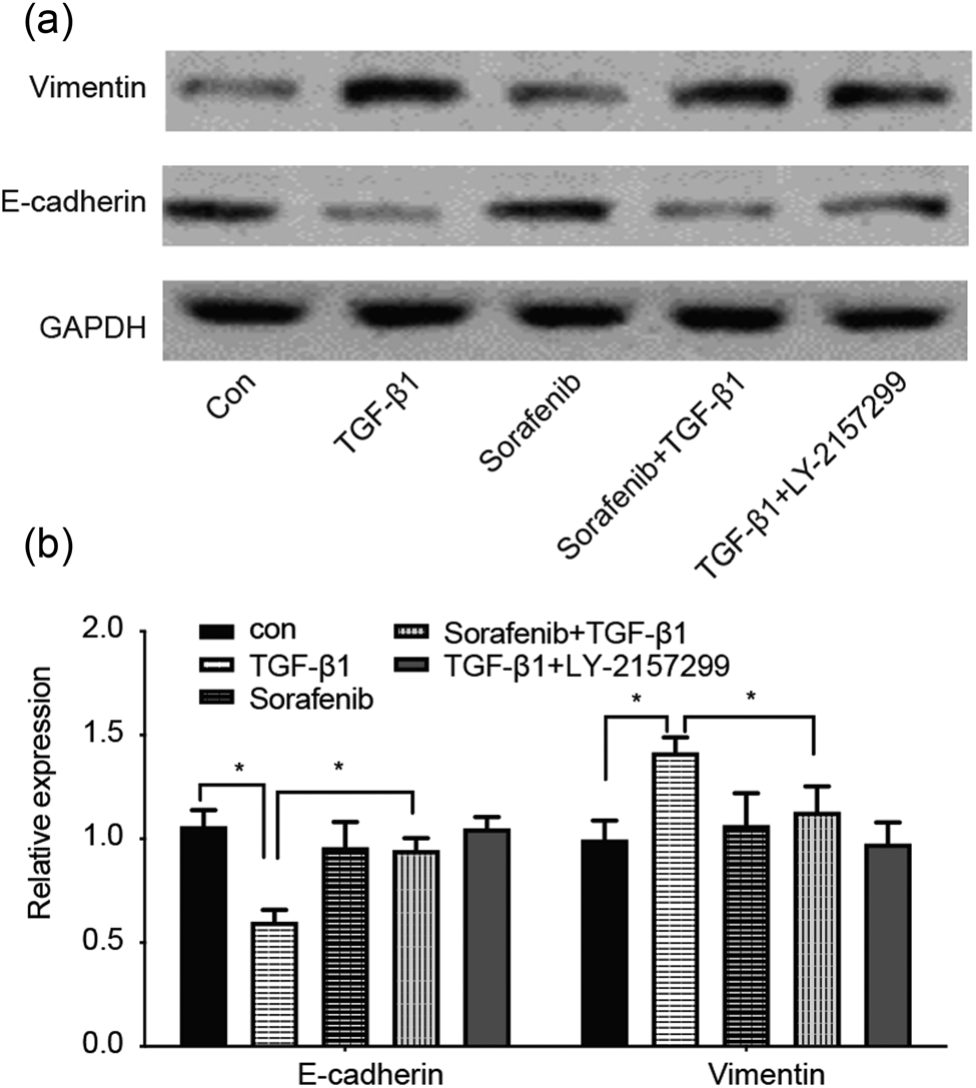
Effects of sorafenib on the regulation of TGF-β1-induced changes in protein expression in ovarian cancer cells. (a) and (b) SKOV-3 cells were cultured and treated in triplicate with 10 µM sorafenib, 10 ng/mL TGF-β1, 10 µM sorafenib + 10 ng/mL TGF-β1, and 10 ng/mL TGF-β1 + 5 µM Ly2157299 for 48 h and then subjected to western blot analysis. The graph shows the quantitative data of these western blots. *P < 0.05.

At the protein level, we assessed the levels of EMT markers (e.g., E-cadherin and vimentin) and found that compared with the control group, the protein expression of E-cadherin in the TGF-β1-treated group of cells was significantly reduced (*P* < 0.05), whereas the protein expression of vimentin was increased (Figure 2(a) and (b)), further confirming that TGF-β1 was able to induce EMT in SKOV3 cells. In contrast, the protein levels of E-cadherin and vimentin were reversed (*P* < 0.05) in the sorafenib + TGF-β1 group and in the TGF-β1 + Ly2157299 group, indicating that LY2157299 (a selective TGF-β1 inhibitor) and sorafenib were able to inhibit TGF-β1-induced SKOV-3 cell EMT *in vitro*.

### 3.2 Effect of sorafenib on reduction in ovarian cancer cell viability

**Figure 3:**
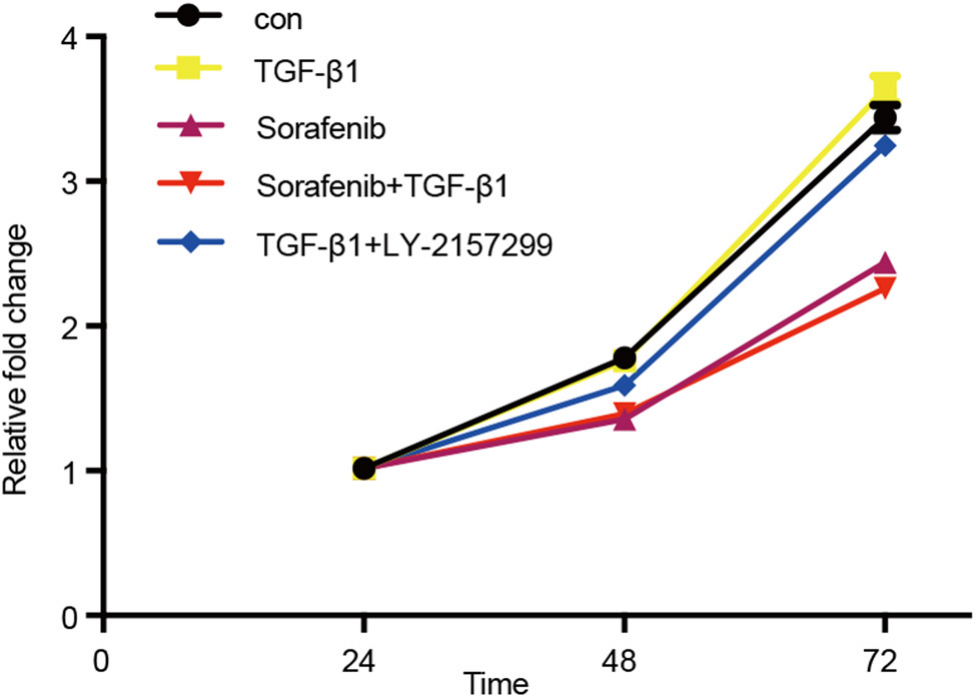
Effects of sorafenib on the downregulation of TGF-β1-induced ovarian cancer cell viability. SKOV-3 cells were cultured and treated in triplicate with 10 µM sorafenib, 10 ng/mL TGF-β1, 10 µM sorafenib + 10 ng/mL TGF-β1, and 10 ng/mL TGF-β1 + 5 µM Ly2157299 for up to 72 h, and a change in cell viability was detected using the MTT assay.

We then performed the cell viability MTT assay and found that there was no significant change in cell viability after TGF-β1-alone or TGF-β1 + LY2157299 treatment (*P* > 0.05), whereas the cell viability after sorafenib-alone or sorafenib + TGF-β1 treatment (*P* < 0.05) was significantly reduced (Figure 3) compared with that of the control group. These findings suggest that TGF-β1 and LY-2157299 had no effect on the SKOV3 cell viability, whereas sorafenib inhibited the tumor cell viability.

### 3.3 Sorafenib activity in the reversal of TGF-β1-induced ovarian cancer cell mobility

**Figure 4:**
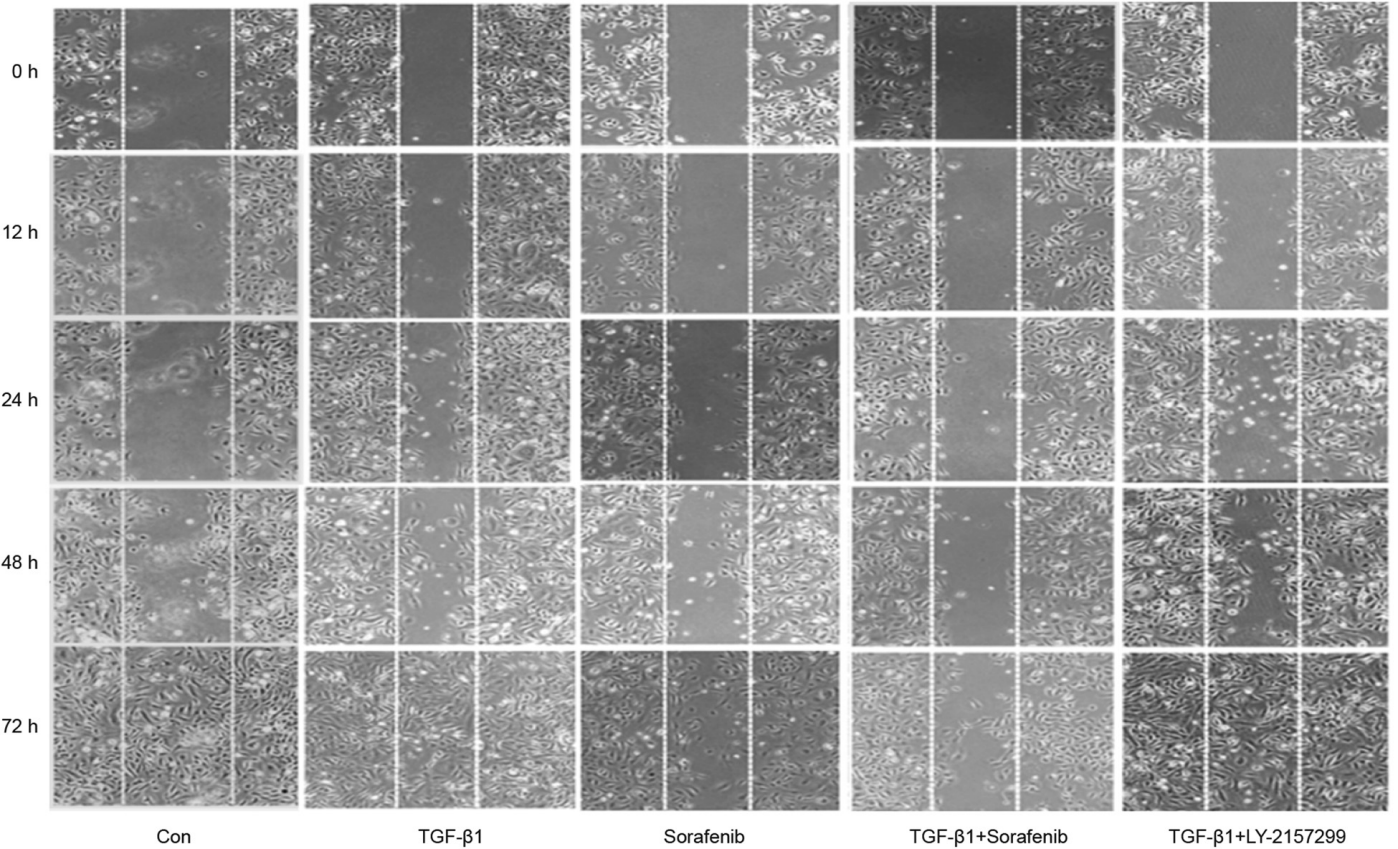
Effects of sorafenib on the downregulation of TGF-β1-induced ovarian cancer cell migration. SKOV-3 cells were cultured and treated in triplicate with 10 µM sorafenib, 10 ng/mL TGF-β1, 10 µM sorafenib + 10 ng/mL TGF-β1, and 10 ng/mL TGF-β1 + 5 µM Ly2157299 for up to 72 h, and the change in cell migration was assessed using a wound-healing assay. The data are summarized in Table 1.

**Figure 5:**
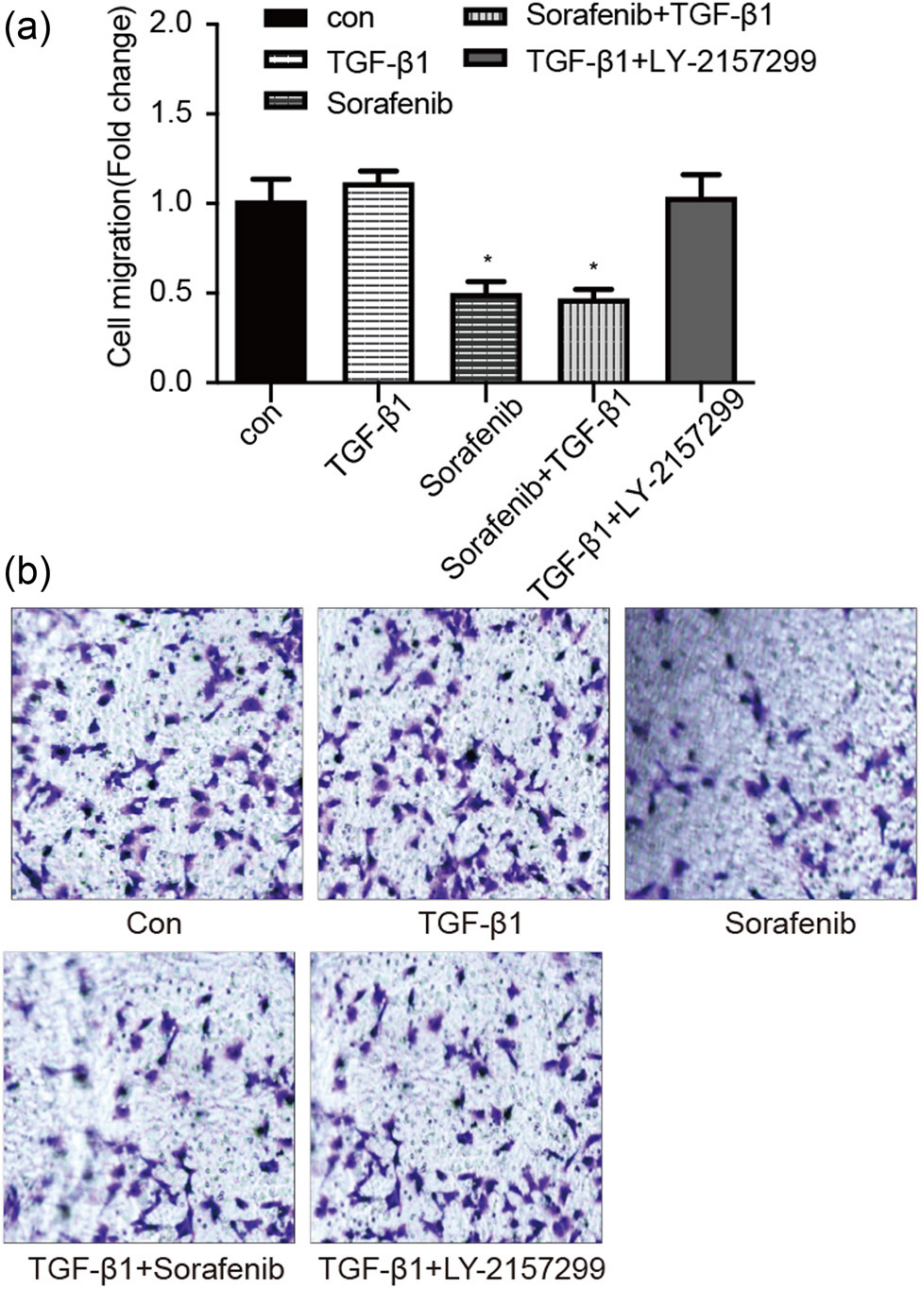
Sorafenib activity in downregulation of TGF-β1-enhanced ovarian cancer cell invasion capacity. (a) and (b) SKOV-3 cells were cultured and treated in triplicate with 10 µM sorafenib, 10 ng/mL TGF-β1, 10 µM sorafenib + 10 ng/mL TGF-β1, and 10 ng/mL TGF-β1 + 5 µM Ly2157299 for 24 h, and the change in cell invasion was assayed using a Transwell assay. *P < 0.05.

Our wound healing assay data showed that the SKOV3 cell migration ability after TGF-β1-alone treatment was obvious, whereas sorafenib-alone or sorafenib + TGF-β1 treatment reduced such an ability (Figure 4 and Table 1). Moreover, our Transwell assay data revealed that there was an enhanced tumor cell invasion capacity after TGF-β1-alone treatment compared with that of the control group. However, sorafenib-alone or sorafenib + TGF-β1 treatment significantly reduced the tumor cell invasion capacity (*P* < 0.05; Figure 5(a) and (b)), suggesting that sorafenib was able to inhibit tumor cell invasion, especially the TGF-β1-enhanced ovarian cancer cell EMT.

**Table 1:** Effect of sorafenib on inhibition of ovarian cancer cell wound healing capacity *in vitro*

Duration (h)	Control	TGF-β1	Sorafenib	TGF-β1 + Sorafenib	TGF-β1 + Ly2157299
0	646.67 ± 15.28	644.33 ± 6.43	645.00 ± 13.23	643.33 ± 22.55	643.33 ± 12.58
12	403.33 ± 20.82	385.00 ± 18.03	591.33 ± 22.59*	623.33 ± 7.64*	505.67 ± 23.00
24	307.00 ± 12.12	293.33 ± 13.58	533.00 ± 25.63*	569.67 ± 17.01*	303.00 ± 16.09
48	228.00 ± 14.73	237.33 ± 11.50	503.67 ± 23.03*	521.00 ± 11.00*	169.67 ± 15.18
72	66.00 ± 20.52	67.33 ± 19.66	450.00 ± 23.07*	472.33 ± 43.55*	97.00 ± 13.89

Note: The data are summarized as the mean value ± standard deviation from Figure 4. *P < 0.001 compared to the control group (*n* = 3).

### 3.4 Sorafenib activity in the reversal of TGF-β1-enhanced ovarian cancer cell radioresistance

**Figure 6:**
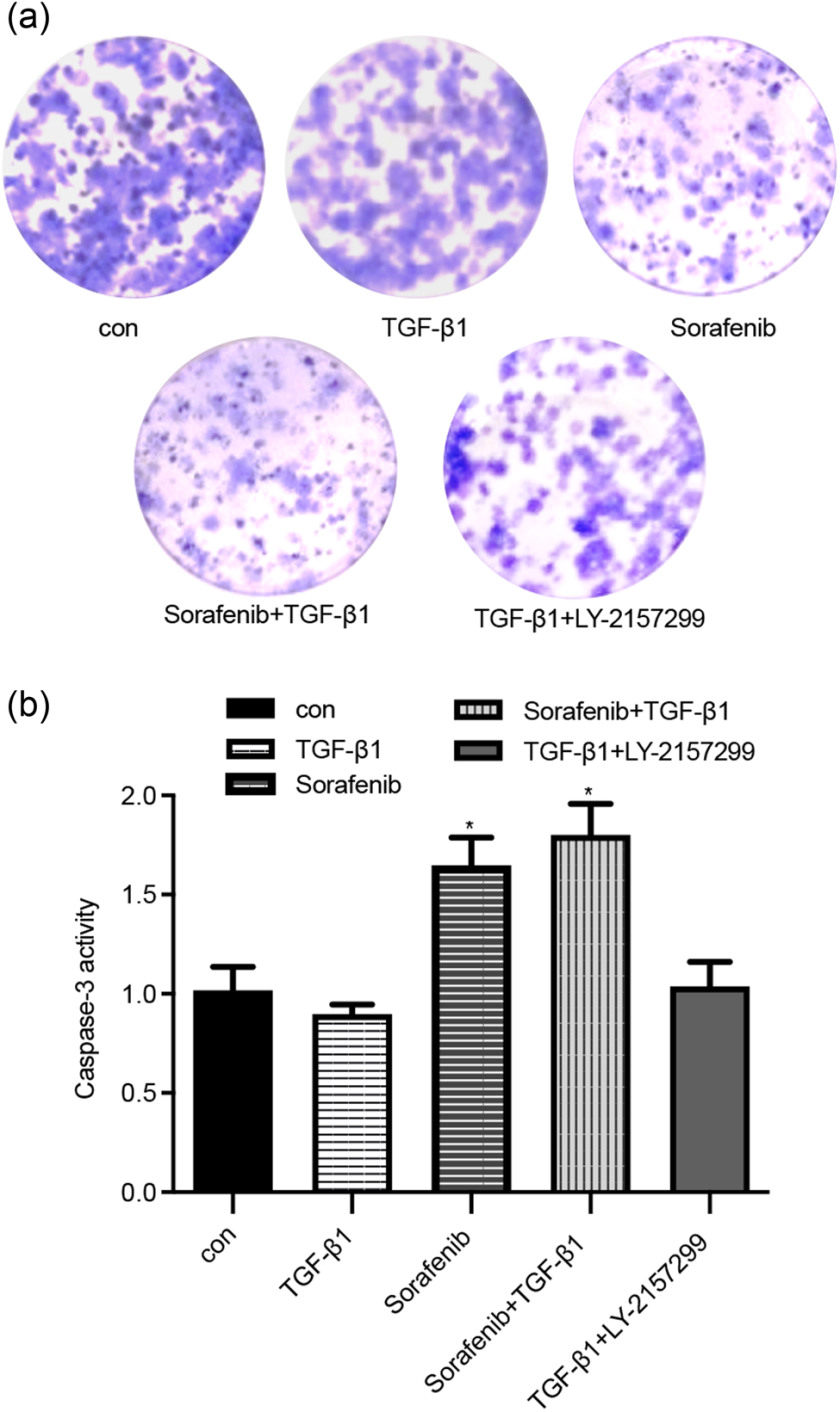
Effects of sorafenib on the upregulation of TGF-β1-reduced ovarian cancer cell radiosensitivity in terms of apoptosis induction. (a) and (b) SKOV-3 cells were grown and treated in triplicate with 10 µM sorafenib, 10 ng/mL TGF-β1, sorafenib (10 µM) + TGF-β1 (10 ng/mL), and TGF-β1 (10 ng/mL) + Ly2157299 (5 µM), respectively, followed by 8-Gy radiation for 8 h, and the radiosensitivity was assayed by using a caspase-3 activity assay. *P < 0.05.

We assayed the caspase-3 activity to determine the level of tumor cell apoptosis. As shown in Figure 6(a) and (b), the activity of caspase-3 in the control group was 1.018 ± 0.118, whereas it was 1.648 ± 0.139 in the sorafenib group and 1.800 ± 0.158 in the sorafenib + TGF-β1 group. The caspase-3 activity was significantly increased after sorafenib-alone or sorafenib + TGF-β1 treatment vs the control group (*P* < 0.05). These data suggest that sorafenib could enhance radiotherapy sensitivity in terms of radiation-induced tumor cell apoptosis.

## 4 Discussion

Ovarian cancer significantly contributes to female mortality worldwide [36,37,38], and the current treatment for ovarian cancer mainly relies on surgery combined with chemotherapy or targeted therapy [6,39]. Although there have been advancements in the treatment of ovarian cancer over the last few decades, the 5-year survival rate of advanced ovarian cancer is still poor, i.e., approximately 30% [40]. In this study, we assessed the antitumor activity of sorafenib in the control of ovarian cancer cell proliferation, mobility, and radiosensitivity *in vitro* using the TGF-β1-enhanced ovarian cancer cell EMT model. As is well known, cytokines play a major role in regulating the cellular responses between tumors and the immune system [41]. Thus, we first confirmed that TGF-β1 treatment was able to enhance the ovarian cancer cell EMT *in vitro* by assessing the tumor cell morphology and expression of EMT markers (E-cadherin and vimentin). In contrast, sorafenib could reverse the TGF-β1-mediated ovarian cancer cell EMT, similar to the effects of Ly2157299, a selective TGF-β1 inhibitor. Sorafenib reduced ovarian cancer cell migration and invasion, but it promoted the radiosensitivity of ovarian cancer. In addition, sorafenib reduced the tumor cell viability. The data from the current study indicate that sorafenib should be tested in clinical trials as a treatment for the control of ovarian cancer progression.

Sorafenib was originally developed and approved for the treatment of advanced renal cell carcinoma and hepatocellular carcinoma [42,43]. The mechanism of action of sorafenib is by inhibiting the activity of various protein kinases, like VEGFR, PDGER, and RAF kinases [44]. and inducing autophagy [45]. In ovarian cancer, sorafenib has been used in combination with various chemotherapeutics and targeting therapeutics, and the results have shown some success in clinical trials [46,47,48,49] as well as a preclinical study [50]. However, contradictory findings have been observed in other clinical trials [51,52,53,54,55], and toxicity has been demonstrated to be an issue [47,48,56]. Thus, further studies are needed to evaluate the activity of sorafenib in ovarian cancer treatment [26,55]. Indeed, another previous clinical study has shown that sorafenib, in combination with topotecan and continued as a maintenance therapy, had a statistically and clinically significant improvement in progression-free survival in platinum-resistant ovarian cancer [50]. Other studies also have revealed that the antiangiogenic therapy did show promise in the treatment of ovarian cancer [57,58]. In our current study, we found that sorafenib suppressed ovarian cancer cell proliferation and mobility by targeting the tumor cell EMT, which is consistent with a previous study [59].

Ovarian cancer invasion and metastasis, like most cancers, are a multi-factorial, multi-step process and are regulated by a variety of genes and gene pathways, although the precise molecular mechanisms are, to date, not fully defined [60,61]. The ovarian cancer cell EMT is an important biological phenomenon that occurs during ovarian cancer development and progression, like all other cancers. During the EMT of tumor cells, the expression of different epithelial markers (e.g., E-cadherin and β-catenin) is reduced, whereas the expression of mesenchymal markers (like fibronectin and vimentin) is induced [62]. Indeed, the EMT was initially discovered and identified in embryonic development, which is essential for embryonic mesenchymal stem cells to differentiate into a variety of cell types and tissues [63]. Nevertheless, the tumor cell EMT leads to cancer development and progression [64–66]. At the gene level, the TGF-β, MAPK, PI3K/AKT, Wnt, and nuclear factor-κB signaling pathways [67] all participate in the tumor cell EMT, and inhibition of the occurrence of the EMT is able to suppress cancer metastasis [68]. Among these signaling pathways, TGF-β is a promoter of tumor invasion and metastasis as it binds to its receptor, phosphorylates the receptor, and, in turn, activates transcription of the target genes and promotes the EMT of cells. To date, the TGF-β1-led signaling pathway is well researched and considered as a classical pathway for induction of the EMT process. It is also true that research on the role of sorafenib in cancer cell EMT has been robust. For example, Nagai *et al*. [69] have demonstrated that sorafenib is able to suppress hepatocyte growth factor-induced liver cancer cell EMT by downregulation of Snail expression through blockage of the RAS/RAF/MEK signaling pathway. In addition, Yuelei et al. [70] have revealed that sorafenib inhibits TGF-β1-induced mouse liver cell EMT by inhibition of TGF-β1-dependent Smad2/3 transcription. In the current study, we first established a cell model of TGF-β1-induced ovarian cancer cell EMT and found that sorafenib was able to increase the protein level of E-cadherin and suppress the protein expression of vimentin, indicating that sorafenib reversed the effect of TGF-β1. Thus, we speculated that sorafenib could be used to effectively control ovarian cancer, especially in combination with other agents or radiation. In the clinic, various antitumor agents, like bevacizumab (a VEGF inhibitor) and sorafenib, have been recognized for their anti-ovarian cancer activity [7,55,57]. Thus, further investigation of the underlying mechanisms of sorafenib could help us to improve the efficacy of this drug against ovarian cancer. It is true that many medications used to treat ovarian cancer patients do possess significant toxicities and adverse effects [71,72,73]. The current study is just a proof-of-principle study, and much more research is needed.

In conclusion, our current study demonstrated that sorafenib was able to reverse the TGF-β1-enhanced ovarian cancer cell EMT and, therefore, suppressed ovarian cancer SKOV-3 cell proliferation and mobility as well as enhanced the radiosensitivity of these cells *in vitro*.
